# Anticipation of Monetary Reward Can Attenuate the Vigilance Decrement

**DOI:** 10.1371/journal.pone.0159741

**Published:** 2016-07-29

**Authors:** Michael Esterman, Mallory Grosso, Guanyu Liu, Alex Mitko, Rachael Morris, Joseph DeGutis

**Affiliations:** 1 Boston Attention and Learning Laboratory, VA Boston Healthcare System, Boston, Massachusetts, United States of America; 2 Geriatric Research Education and Clinical Center (GRECC), Boston Division VA Healthcare System, Boston, Massachusetts, United States of America; 3 Department of Psychiatry, Boston University School of Medicine, Boston, Massachusetts, United States of America; 4 Department of Psychiatry, Harvard Medical School, Boston, Massachusetts, United States of America; 5 School of Psychology, University of Kent, Canterbury, United Kingdom; University of Verona, ITALY

## Abstract

Motivation and reward can have differential effects on separate aspects of sustained attention. We previously demonstrated that continuous reward/punishment throughout a sustained attention task improves overall performance, but not vigilance decrements. One interpretation of these findings is that vigilance decrements are due to resource depletion, which is not overcome by increasing overall motivation. However, an alternative explanation is that as one performs a continuously rewarded task there are less potential gains/losses as the task progresses, which could decrease motivation over time, producing a vigilance decrement. This would predict that keeping future gains/losses consistent throughout the task would reduce the vigilance decrement. In the current study, we examined this possibility by comparing two versions (continuous-small loss vs. anticipate-large loss) of a 10-minute gradual onset continuous performance task (gradCPT), a challenging go/no-go sustained attention task. Participants began each task with the potential to keep $18. In the continuous-small-loss version, small monetary losses were accrued continuously throughout the task for each error. However, in the anticipate-large-loss version, participants lost all $18 if they erroneously responded to one target that always appeared toward the end of the vigil. Typical vigilance decrements were observed in the continuous-small-loss condition. In the anticipate-large-loss condition, vigilance decrements were reduced, particularly when the anticipate-large loss condition was completed second. This suggests that the looming possibility of a large loss can attenuate the vigilance decrement and that this attenuation may occur most consistently after sufficient task experience. We discuss these results in the context of current theories of sustained attention.

## Introduction

Sustaining attention over time is critical to everyday activities such as driving, reading, and listening to a lecture. Although it is imperative for many tasks, remaining focused is challenging and people often experience lapses of attention [1]. These attentional failures can lead to real world incidents such as automobile or train accidents [2, 3].

Two prominent theories have been employed to explain sustained attention failures. On the one hand, the mindlessness theory proposes that failures in sustaining attention are due to underarousal, boredom, or disinterest—factors that can be affected by reward and motivation. More specifically, the mindlessness theory attributes attention failure to suboptimal engagement of the supervisory attentional system to direct attention to tasks as they become monotonous [4, 5]. In support of this perspective, task-unrelated thoughts, mind wandering, and related physiological and neural systems are associated with sustained attention failures (e.g., [6–9]). As motivation and reward have been shown to enhance engagement of these attentional systems (e.g., [10, 11]), the mindlessness theory predicts that these factors should enhance sustained attention.

On the other hand, resource models propose that attentional failures, particularly those that increase over time (i.e., vigilance decrement), are due to the depletion of a limited pool of attentional resources that require breaks or rest to replenish [12, 13]. In support of this perspective, increasing demands on attentional resources with factors such as novelty, stimulus degradation, or working memory demands, elicit greater attention failures and vigilance decrements [13–16] and further, such decrements are associated with less neural activation in attention-related brain regions [17]. To the extent that reward and motivation engage these attentional and working memory resources, this theory predicts that if anything, it could accelerate the depletion of the resources.

We recently used reward-based motivation during sustained attention to test these two theoretical predictions [18]. We found that reward enhanced overall performance, but had no effect on the vigilance decrement. This supports a hybrid model in which overall performance is impacted by mindlessness (e.g., underarousal) and is improved with reward, while the vigilance decrement is due to resource depletion and is not affected by reward. This is consistent with other work suggesting an important dissociation between overall performance and the vigilance decrement [19, 20]. In fact, [19] suggested, “The mindlessness model has some merit in regards to understanding overall signal detection rates, but not in accounting for the vigilance decrement” (p. 23). Our own work has found that attention failures can be precipitated by both “mindlessness” (as indexed by high default mode activity) and “depletion” (as indexed by low attention network activity) and that these depletion errors are more likely to occur later in the vigil [21]. Taken together, attempts to resolve the two theoretical accounts suggest that while some failures to engage attention are due to suboptimal motivation, other failures could be due to attentional resources being depleted or unavailable.

Though our recent study, along with a wealth of data (e.g., [18,19,22]) are consistent with the vigilance decrement being accounted for by resource theory, a recent alternative model has been proposed. The opportunity cost model [23] is a *non-resource* account that posits that performing mental tasks engages cognitive mechanisms (e.g., working memory or attention systems) that can only be deployed on a limited number of tasks, but nevertheless do not get “used up”. Hence, the deployment of these mechanisms carries an opportunity cost: the value of alternatives to the current task. When the value of alternatives exceeds the value of the current task, one will experience an aversive feeling of mental effort, which motivates one to reallocate the mechanisms to the alternatives. Thus, the theory does not attribute performance decline to dwindling resources, but attributes it to a redistribution of cognitive resources. A variant of this theory, resource control theory, suggests that this redistribution of cognitive resources is specific to executive control, and that subjects “learn” to adaptively reduce this control for a preferred default state characterized by mind-wandering [24] (also see [25]). Although a newer theory, some recent data supports this opportunity cost perspective. In particular, Hopstaken and colleagues [26] found that time-on-task over a period of two hours lead to vigilance decrements and reduced stimulus-evoked pupillary dilations (a measure of arousal). Interestingly, after two hours, performance was incentivized and both performance and stimulus-evoked pupil dilation recovered, suggesting that additional task resources were still available after such an extended period of performance. In addition, our recent findings of normal vigilance decrements in the context of high motivation and reward could also be consistent with this opportunity cost model. Specifically, it may be that subjects gradually shifted attention away from the current task to other tasks (e.g., introspection, future planning) as the amount of time left in the task/potential money reward decreased, i.e., as the opportunity cost of engaging the attention systems in the task gradually increased.

One potential way to adjudicate between the resource vs. opportunity cost models is to keep potential gains/losses constant throughout the entire task, and thus keep the opportunity cost constant. Resource theory would predict that even if the motivation to perform the task remains constant over time, there would continue to be a depletion of attentional resources over time resulting in a vigilance decrement. In contrast, the opportunity cost model predicts that keeping the opportunity cost constant throughout the task should reduce the vigilance decrement: subjects should *not* reallocate their attention away from the current task over time.

In the current study, we examined this possibility by comparing two versions (continuous-small loss vs. anticipate-large loss) of the gradual onset continuous performance task (gradCPT), a challenging go/no-go sustained attention task, in which participants were instructed to respond to frequent city scenes and inhibit responses to infrequent mountain scenes. The gradCPT is a reliable tool for measuring sustained attention ability and eliciting vigilance decrements in short durations, and due to its gradual nature/reduced abrupt onsets, relies more heavily on endogenous attentional control [21, 27, 28]. Each gradCPT began with the potential to keep an $18 reward. In the continuous-small-loss version, participants would lose $0.25 of the reward for each mountain they erroneously pressed to, and would keep $0.25 for each mountain in which responses were correctly inhibited, such that losses were accrued continuously throughout the task (with a total of $18 at stake). In contrast, in the anticipate-large-loss version, participants would lose all $18 if they pressed to the one target mountain, which was briefly outlined in green so participants were aware of its presence and their accuracy. While they were told this single $18 mountain trial would occur randomly, it always occurred after 10-minutes. Thus, up until that point, the possible gain or loss of $18 remained constant. While the continuous-loss condition should replicate a reliable and comparable vigilance decrement as our previous work, the anticipate-loss condition was designed to differentiate between the opportunity cost and resource accounts of the vigilance decrement.

## Methods: Experiment 1

### Participants

Thirty-six participants recruited from local universities (20 females) ages 18–28 (M = 20.89, SD = 2.67) performed two versions (continuous-loss and anticipate-loss) of the gradual onset continuous performance task (gradCPT). The study was approved by the VA Boston Healthcare System IRB, and written consent was obtained from all participants. Note that sample sizes were chosen based on measuring the vigilance decrement in our prior work and power analysis [18]. Specifically, our formal power analysis was conducted in order to determine what sample size would be likely required to detect differences in the vigilance decrement. To do this, we identified 5 papers in the literature that explored experimental manipulations of vigilance decrements [19, 22, 29–31]. The mean effect size (d) across these five experiments was d = 1.18. Detecting this effect at 95% power required 17 subjects. We used this power analysis to motivate our previous study [18] and the current study, recruiting 18 participants in all experiments. Based on this, to the best of our knowledge, the current set of experiments was powered to detect dissociations between the continuous-small and anticipate-large conditions on gradCPT vigilance decrements. Also note that in all subsequent between-subject (cross-experiment) analyses, no differences in age or gender were present.

### Procedure

Before beginning the gradual onset continuous performance task (gradCPT, see below; [18, 21, 27]), participants were familiarized with the tasks and were given 1.5–3 minutes of practice. After completing the first gradCPT and before the second gradCPT, participants filled out a series of questionnaires for ~20 minutes (data not reported).

In Experiment 1a, participants completed the continuous-small-loss task, followed by the anticipate-large-loss task (first 18 subjects). In Experiment 1b, the order of tasks was reversed (the second set of 18 subjects).

### GradCPT

The gradual onset continuous performance task (gradCPT; [18, 21, 27]) is a continuous performance task consisting of 20 round, grayscale photographs of 10 city scenes and 10 mountain scenes. The scenes were presented randomly (90% city and 10% mountain) without two consecutive trials being identical. The scenes gradually transitioned from one to the next every ~800 ms using a linear pixel-by-pixel interpolation. Participants were instructed to respond to frequently occurring nontargets (city scenes) and withhold a response to the infrequent targets (mountain scenes). Response accuracy was emphasized without reference to speed. However, given that the next stimulus would replace the current stimulus in ~800 ms, a response deadline was implicit in the task. As in previous work, our primary measures of sustained attention ability were accuracy (d’) and reaction time variability (coefficient of variation, CV), reflecting the ability to discrimination cities/mountains and maintain attentional stability, respectively [27]. We used two versions of the gradCPT in this experiment (see below).

### Continuous-small-loss version

Participants began this task with the potential to keep $18. Participants were told that they would lose $0.25 for each commission error they made (pressing to a mountain), such that losses were accrued continuously throughout the task. They were also made aware that even if they correctly withheld their response to a mountain, they would also lose $.25 if they did not press to the preceding city scene. This was done to ensure participants did not simply withhold correct responses in order to maximize reward. The task lasted 10 minutes and upon completion, a screen displayed the reward amount that the participant retained based on their actual performance.

### Anticipate-large-loss version

Similar to the continuous-small-loss version, participants began this task with the potential to keep $18. Participants were familiarized with a sample target high-reward mountain and told that they would lose all $18 if they pressed to this mountain, which was briefly outlined in green during the task so that participants were aware of its presence and their accuracy. Note that the high-reward mountain trial could be any of the 10 mountain images (randomly selected), and was only identifiable by the green border. Again, they were also instructed that even if they correctly withheld their response to the target mountain, they would also lose $18 if they did not press to the preceding city scene. Although participants were told this single, large-loss trial would occur randomly, it always occurred after 10 minutes (the task was 12 minutes in total). Only the 10 minutes of data preceding the target mountain were analyzed, as a direct comparison to the continuous-small-loss version (10 minutes).

### Comparison Results

To better understand the effects of reward/motivation on gradCPT in the current study relative to other rewarded and unrewarded versions of the gradCPT, we compared the results of this study to [18], which conducted a series of rewarded and unrewarded experiments using similar tasks. In this previous study, participants were assigned to one of four conditions as follows: 1) no reward condition 2) time reward, in which participants were told that the length of the task would be directly related to their performance 3) a performance-based monetary reward condition similar to the continuous-small-loss condition in the current study and 4) performance-based monetary reward condition where feedback on the amount accumulated was presented periodically. The results from this study showed that when participants were rewarded (conditions 2–4), their overall performance (d’ and RT variability) was significantly more accurate and less variable than those in the no-reward condition (condition 1). However, participants in all four conditions still showed significant and statistically indistinguishable vigilance decrements in d’ and CV. Thus, these prior experiments serve as a comparison to both replicate the effect in our continuous-small-loss condition, and contrast several other types of incremental reward (and no reward) with the more novel anticipate-large-loss condition.

### Analysis

#### Accuracy

Trials in which participants correctly inhibited a button press to mountain scenes were considered hits (correct omissions). Trials in which participants erroneously responded to mountains were considered misses (commission errors). Trials in which participants failed to respond to city scenes were considered false alarms (omission errors). D’ was calculated as a measure of accuracy that incorporated both types of trials.

#### Reaction time

RTs were calculated relative to the beginning of each image transition, such that an RT of 800 ms indicates a button press at the moment image n was 100% coherent and not mixed with other images. A shorter RT indicates that the current scene was still in the process of transitioning from the previous, and a longer RT indicates that the current scene was in the process of transitioning to the subsequent scene. For example, an RT of 720 ms would be at the moment of 90% image n and 10% image n − 1. On rare trials with highly deviant RTs (before 70% coherence of image n and after 40% coherence of image n + 1) or multiple button presses, an iterative algorithm maximized correct responses as follows: The algorithm first assigned unambiguous correct responses, leaving few ambiguous button presses (presses before 70% coherence of the current scene and after 40% coherence of the following scene). Second, ambiguous presses were assigned to an adjacent trial if one of the two had no response. If both adjacent trials had no response, the press was assigned to the closest trial, unless one was a no-go target, in which case subjects were given the benefit of the doubt that they correctly omitted. Slight variations to this algorithm yielded highly similar results, as most button presses showed a 1–1 correspondence with presented images. Raw RTs on correct trials were used to calculate RT variability, or coefficient of variation (CV = standard deviation of RT/meanRT).

#### Time-on-task effects

Identical to our previous study [18], time-on-task effects, or vigilance decrements, were measured by calculating 2-min windows around RT variability and accuracy measures of interest (CV and d’), where the first window included 0–2 min and the last included 8–10 min. Linear slopes were calculated with these 5 quintiles (computed as rate of change per minute) for each subject and task. T-tests were used to contrast slopes with 0 (to determine whether there was significant linear change over time), as well as to compare slopes across experiments.

#### Subject outliers

When pooled across all experiments (1a, 1b, 2, and 3), those participants who performed outside of three standard deviations from the mean on any of the main dependent measures (CV and d’, overall and slopes) were excluded. Notably, we used the identical procedure as our previous gradCPT/reward study [18]. This resulted in the elimination of only 1 (out of 72 total) participant, who took part in Experiment 1b.

## Results: Experiment 1

### Overall performance: Experiment 1a and 1b

#### Accuracy

**Fig 1:**
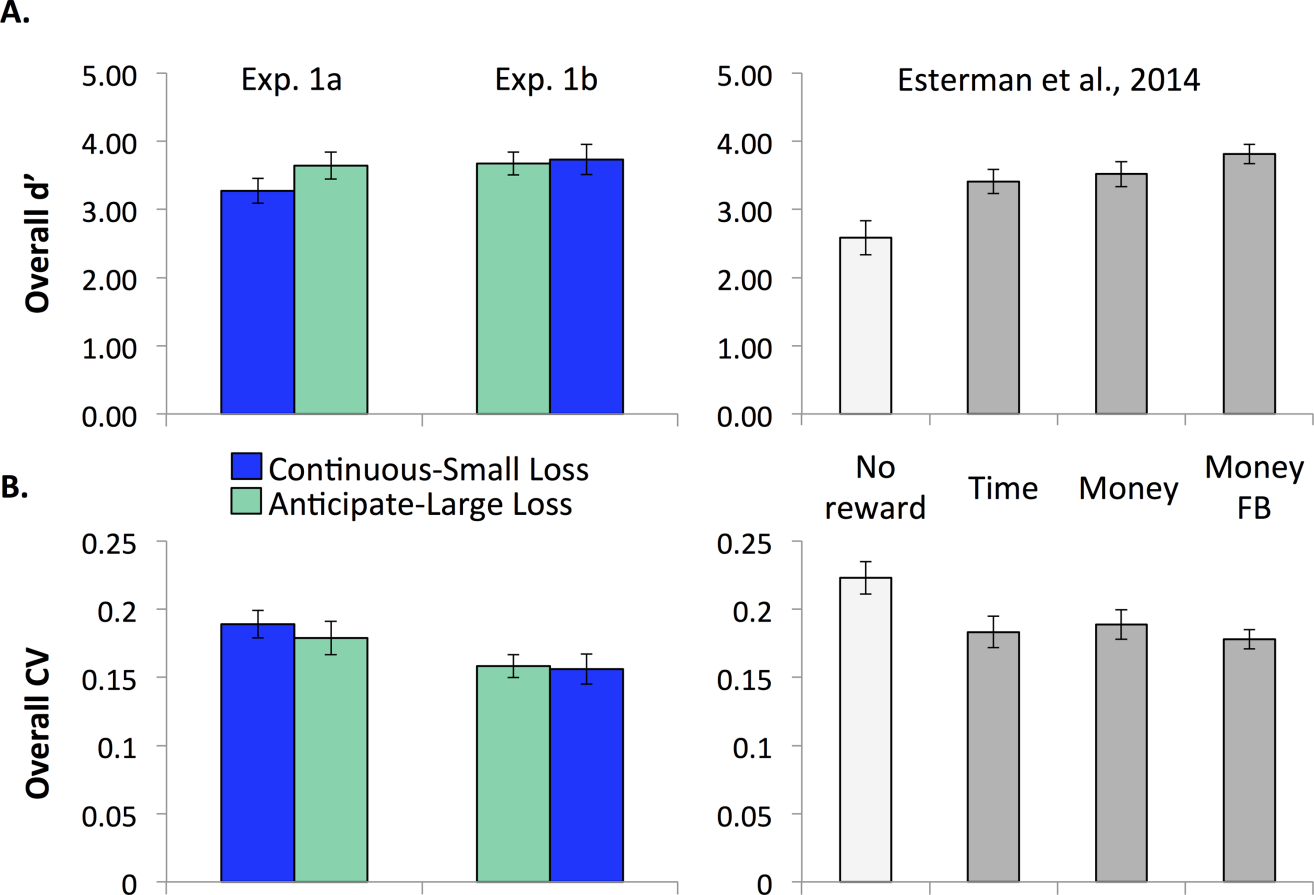
Overall performance in Experiment 1 and comparison to [18]. A. Overall accuracy (d’). B. Overall RT variability (CV). In both measures, rewarded performance exceeds non-rewarded performance (higher d’ and lower CV).

We first sought to confirm that rewarded performance in the continuous-small and anticipated-large-loss conditions was better than the non-rewarded condition from [18]. As can be seen in Fig 1A, performance in both Experiment 1a and 1b for continuous-small loss was significantly higher than participants performing a non-rewarded version of the identical task in [18] (Experiment 1a: t(34) = 2.21, *p* = 0.034, η^2^ = .13; Experiment 1b: t(33) = 3.38, *p* = 0.002, η^2^ = .26). Similarly, for the anticipate-large-loss task, overall *d’* was significantly higher than the non-rewarded condition in [18] (Experiment 1a: t(34) = 3.28, *p* = 0.002, η^2^ = .24; Experiment 1b: t(33) = 3.59, *p* = 0.001, η^2^ = .28).

We next sought to determine if the current reward structure had similar effects as the rewarded conditions in [18]. A one-way ANOVA showed that there was no difference in overall *d’* performance for all of the reward conditions in the current study and in [18], regardless of which task was done first (ANOVA with continuous-small loss 1a, anticipate-large loss 1b, [18] rewarded: F(4, 82) = 1.586, *p* = 0.186; ANOVA with anticipate-large loss 1a, continuous-small loss 1b, [18] rewarded: F(4, 82) = 0.732, *p* = 0.573). Thus, the current rewards had equivalent effects on overall accuracy as previous studies.

#### Reaction time variability

Similar patterns were observed in reaction time variability. As can be seen in Fig 1B, participants’ overall RT variability for the continuous-small-loss task was significantly lower compared to the non-rewarded group in [18] (Experiment 1a: t(34) = -2.18, *p* = 0.036, η^2^ = .12; Experiment 1b: t(33) = -4.12, *p*<0.001, η^2^ = .34). These significant differences also were present for the anticipate-large-loss task (Experiment 1a: t(34) = -2.58, *p* = 0.014, η^2^ = .16; Experiment 1b: t(33) = -4.40, *p*<0.001, η^2^ = .37).

In comparison to previous rewarded conditions [18], one-way ANOVAs showed no differences between CV in the current rewarded and previously rewarded gradCPTs (ANOVA with continuous-small loss 1a, anticipate-large loss 1b, [18] rewarded: F(4, 82) = 1.720, *p* = 0.153; ANOVA with anticipate-large loss 1a, continuous-small loss 1b, [18] rewarded: F(4, 82) = 1.353, *p* = 0.257). Thus, the current rewards had equivalent effects on overall response time variability as previous studies.

#### Continuous-small loss vs. anticipate-large loss

To determine if there were overall performance differences between continuous-small loss and anticipate-large loss, and whether this interacted with order, we first compared overall performance between Experiment 1a and 1b using a mixed ANOVA (order and condition as factors). This revealed that there was no significant main effect or interaction with order (*d’*: main effect, F(1, 33) = 1.827, *p* = .186; interaction, F(1, 33) = 3.447, *p* = 0.072; RT variability: main effect, F(1, 33) = 1.040, *p* = 0.315; interaction, F(1, 33) = 2.522, *p* = 0.122).

Taken together, these results show that the reward manipulations in the current study had the similar enhancing effect on *overall* accuracy and *overall* response variability as the rewarded conditions in [18], and overall performance was consistent across order of completion.

### Time-on-task effects: Experiment 1a

#### Accuracy

**Fig 2:**
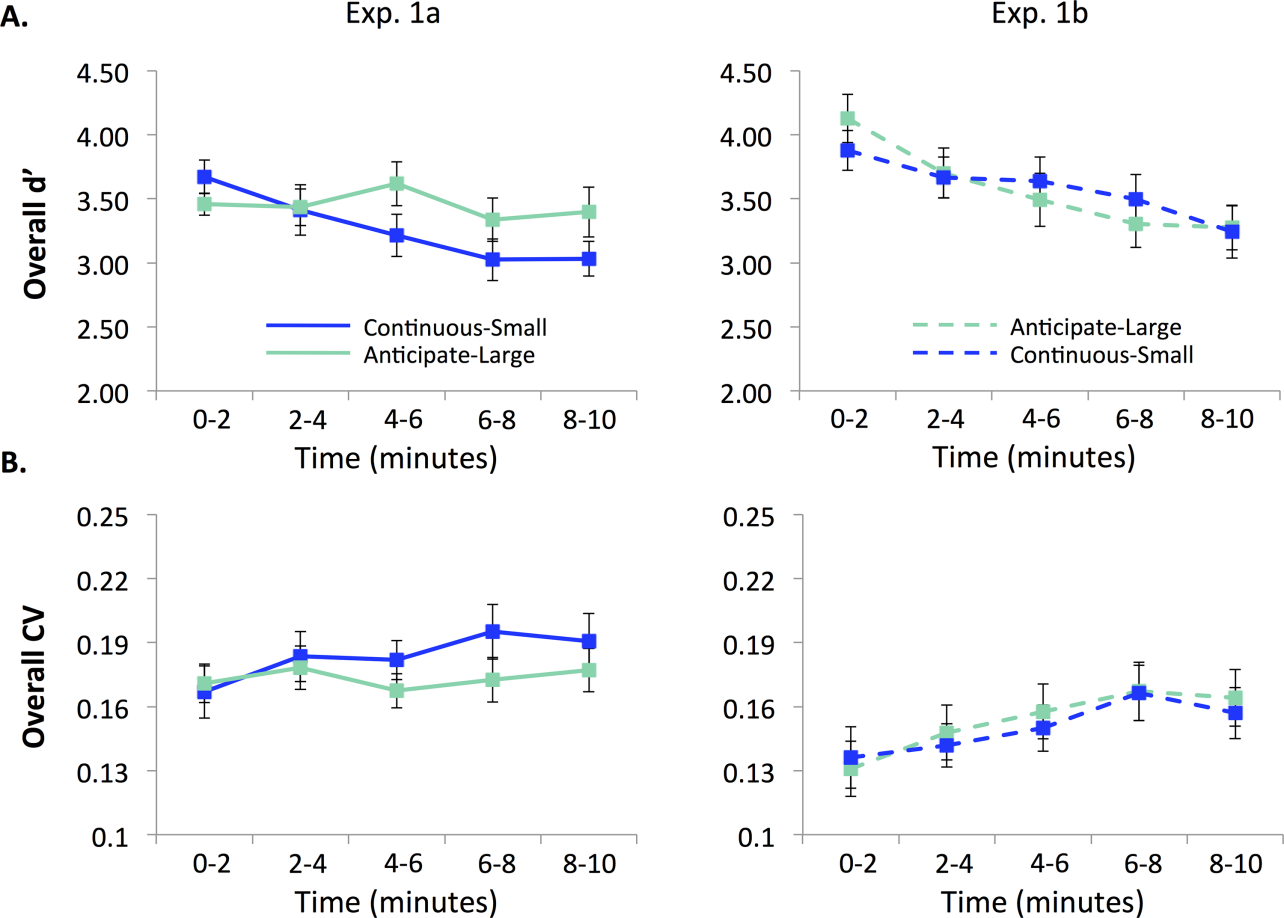
**Performance in Experiment 1a (left) and 1b (right) divided into 2-min quintiles.** A. Accuracy (d’) and B. RT variability (CV). All task versions exhibited performance decrements over time (lower d’ and higher CV) with the exception of the Anticipate-large-loss task in Experiment 1a.

**Fig 3:**
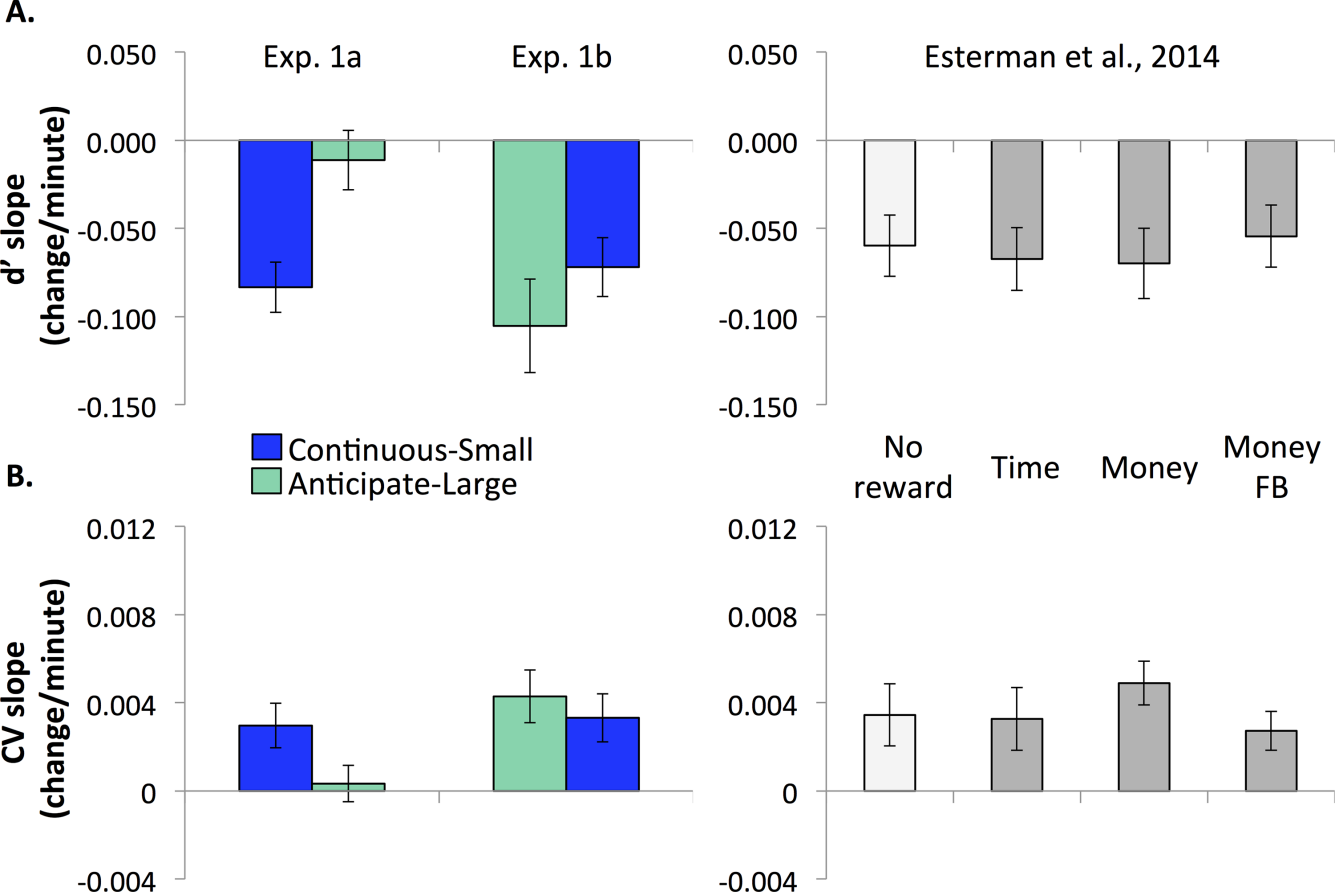
Vigilance decrements, or linear changes over time, in Experiment 1. Linear slopes were computed from quintile data (Fig 2) for A. Accuracy (d’) and B. RT variability (CV). Decrements/slopes from [18] are displayed for comparison. All versions exhibited comparable performance decrements over time (lower d’ and higher CV) with the exception of the Anticipate-large-loss task in Experiment 1a.

Time-on-task effects in accuracy differed between the first task, continuous-small loss, and the second task, anticipate-large loss in Experiment 1a (Figs 2A and 3A). During the continuous-small-loss task, participants exhibited accuracy decrements (M = -0.08 d’/min, SD = 0.06; significantly different from 0, t(17) = -5.852, *p* < .001, η^2^ = .67). Furthermore, this slope was not significantly different from all rewarded subjects in [18] (t(68) = -0.990, *p* = 0.326, η^2^ = .014). In contrast, when performing the second task, anticipate-large loss, participants did not show a significant accuracy decrement (M = -0.01, SD = 0.07; not significantly different from 0, t(17) = -0.667, *p* = 0.514, η^2^ = .025). This slope was significantly different from the continuous-small-loss slope (t(34) = -3.271, *p* = 0.002, η^2^ = .24), as well as from all rewarded subjects in [18] (t(68) = 2.565, p = .013, η^2^ = 0.088).

#### Reaction time variability

The same patterns were observed in RT variability (Figs 2B and 3B). During the continuous-small-loss task, participants exhibited significant performance decrements in CV (M = 0.003, SD = 0.004; significantly different from 0, t(17) = 2.941, *p* = .009, η^2^ = .34). Again, this slope was not significantly different from all rewarded subjects in [18] (t(68) = -0.55, *p* = 0.584, η^2^ = .0044). However, when performing the anticipate-large-loss task, participants did not show a significant decrement (M = 0.0003, SD = 0.003; not significantly different from 0, t(17) = 0.406, *p* = 0.69, η^2^ = .0096). This slope was marginally different from the continuous-small-loss slope (t(34) = 2.027, *p* = .05, η^2^ = .11), as well as significantly different from all rewarded subjects in [18] (t(68) = -2.795, *p* = 0.007, η^2^ = .10).

These results demonstrate that when participants performed the continuous-small-loss task first, they had a vigilance decrement similar to what has been found in previous research. However, when participants performed the anticipate-large-loss task second, they did not show a vigilance decrement in accuracy or variability and were able to maintain consistent performance throughout the 10-minute of task.

### Time-on-task effects: Experiment 1b

#### Accuracy

In contrast to Experiment 1a, Experiment 1b did not reveal differences in time-on-task effects between the anticipate-large and continuous-small-loss tasks (Figs 2A and 3A). Similarly to Experiment 1a, when participants performed the continuous-small-loss task (second task) they had significant accuracy decrements (M = -0.07, SD = 0.07; significantly different from 0, t(16) = -4.192, *p* = 0.001, η^2^ = .52). This slope was not significantly different from all rewarded subjects in [18] (t(67) = -0.392, *p* = 0.696, η^2^ = .0023). However, unlike Experiment 1a, in Experiment 1b participants also showed a significant decrement when performing the anticipate-large-loss task first (M = -0.105, SD = 0.112; significantly different from 0, t(16) = -3.861, *p* = 0.001, η^2^ = .48). This slope was not significantly different from all rewarded subjects in [18] (t(67) = -1.421, *p* = 0.170, η^2^ = .029).

#### Reaction time variability

The same time-on-task patterns for accuracy were also observed in RT variability (Figs 2B and 3B). During the continuous-small-loss task, participants had a significant decrement in RT variability (M = 0.003, SD = 0.005; significantly different from 0, t(16) = 2.955, *p* = 0.009, η^2^ = .35). Again, this slope was not significantly different from the rewarded participants in [18] (t(67) = -0.252, *p* = 0.802, η^2^ = .00095). Similar to above, there was also a significant decrement in RT variability during the anticipate-large-loss task (M = 0.004, SD = 0.005; significantly different from 0, t(16) = 3.479, *p* = 0.003, η^2^ = .43). This was also not significantly different from all rewarded participants in [18] (t(67) = 0.489, *p* = 0.626, η^2^ = .0036).

Thus, in summary, Experiment 1b revealed that order of the anticipate-large-loss task was a critical factor determining time-on-task effects. When performed first (Experiment 1b), a consistent decrement was observed in discrimination ability and variability that matched all prior versions and experiments. In contrast, when performed second (Experiment 1a), participants were able to maintain performance and have no discernable decrement.

### Time-on-task effects: Experiment 1a vs. 1b

To more formally test for this task-order interaction between the Experiment 1a and 1b, we conducted a mixed ANOVA with task (continuous-small loss and anticipate-large loss) and order (1a vs. 1b) as factors. This analysis showed that there was an interaction between task and order (F(1, 33) = 11.44, *p* = 0.002). This pattern was similar for RT variability slope such that the interaction trended towards significance (F(1, 33) = 3.731, *p* = 0.062).

These results further confirm that whether there is a vigilance decrement in the anticipate-large-loss task is determined by the order in which it is performed. One explanation for the lack of vigilance decrement in the anticipate-large-loss condition when performed second, but not first, is that the vigilance decrement can only be eliminated with constant motivation/reward once the task is sufficiently practiced. This may be because working memory demands for a novel task may be greater (e.g., [32]). Thus when performed first, there is resource depletion for this high-load task, whereas after experience, the load is reduced, allowing motivational factors to reduce the decrement. Importantly, these motivational factors only reduce the decrement when they are held constant (anticipate-large-loss task), as the decrement is comparable in the continuous-small-loss task regardless of order. A second possible explanation is that the order of the tasks provides a specific type of context that may influence performance. For example, in Experiment 1a, the first task consists of many trials with small stakes, while the second task consists of a single large stake trial. This difference in reward type could cause the second task to be more arousing or motivating, particularly in the context of having performed the first. In contrast, in Experiment 1b, the anticipate-large-loss task is performed without the prior context of many trials with small stakes.

To tease apart these alternatives we ran two follow-up experiments that varied the context of the first gradCPT version to the two extremes, followed by the anticipate-large-loss task. First, in Experiment 2, we sought to replicate the results of Experiment 1a, but rather than starting with small rewards, we eliminated reward entirely in the first gradCPT, followed by the anticipate-large-loss version. Thus, whether due to practice or context, Exp. 2 should simply replicate and confirm that when performed second, the anticipate-large-loss condition eliminates the vigilance decrement. To then eliminate the effects of context, in Experiment 3 participants performed the anticipate-large loss twice in order to keep reward context constant throughout the experiment, and thus remove any effects of starting with smaller rewards, or less arousing reward context. Thus, if the effect in Experiment 1a (and Exp. 2) was due to prior context of smaller-losses, we should see a vigilance decrement in both iterations of the anticipate-large-loss task. On the other hand, if the attenuated vigilance decrement was due to practice, we should see a decrement in the first anticipate-large-loss task, but not the second.

## Methods: Experiment 2

### Participants

Eighteen participants were recruited from two local universities (same as Experiment 1) (10 females) ages 18–25 (M = 21.39, SD = 2.09) and performed a non-rewarded (“practice”) gradCPT task followed by the anticipate-large-loss task. The study was approved by the VA Boston Healthcare System IRB, and written consent was obtained from all participants.

### Procedure

This experiment was similar to Experiment 1 except that participants completed a non-reward version of the gradCPT followed by the anticipate-large-loss version of gradCPT.

## Results: Experiment 2

### Overall performance

#### Accuracy

The overall accuracy (d’) for the non-rewarded condition was 2.87 (SD = 0.849) and 3.76 (SD = 1.06) for anticipate-large loss. As expected, these conditions were significantly different from each other (t(34) = -2.80, *p* = 0.008, η^2^ = .19).

#### Reaction time variability

The overall RT variability (CV) in the non-rewarded task was 0.217 (SD = 0.0448) and 0.184 (SD = 0.0579) for the anticipate-large-loss task. This difference between the two tasks was marginally significant (t(34) = 1.885, *p* = 0.068, η^2^ = .095).

### Time-on-task effects

#### Accuracy

**Fig 4:**
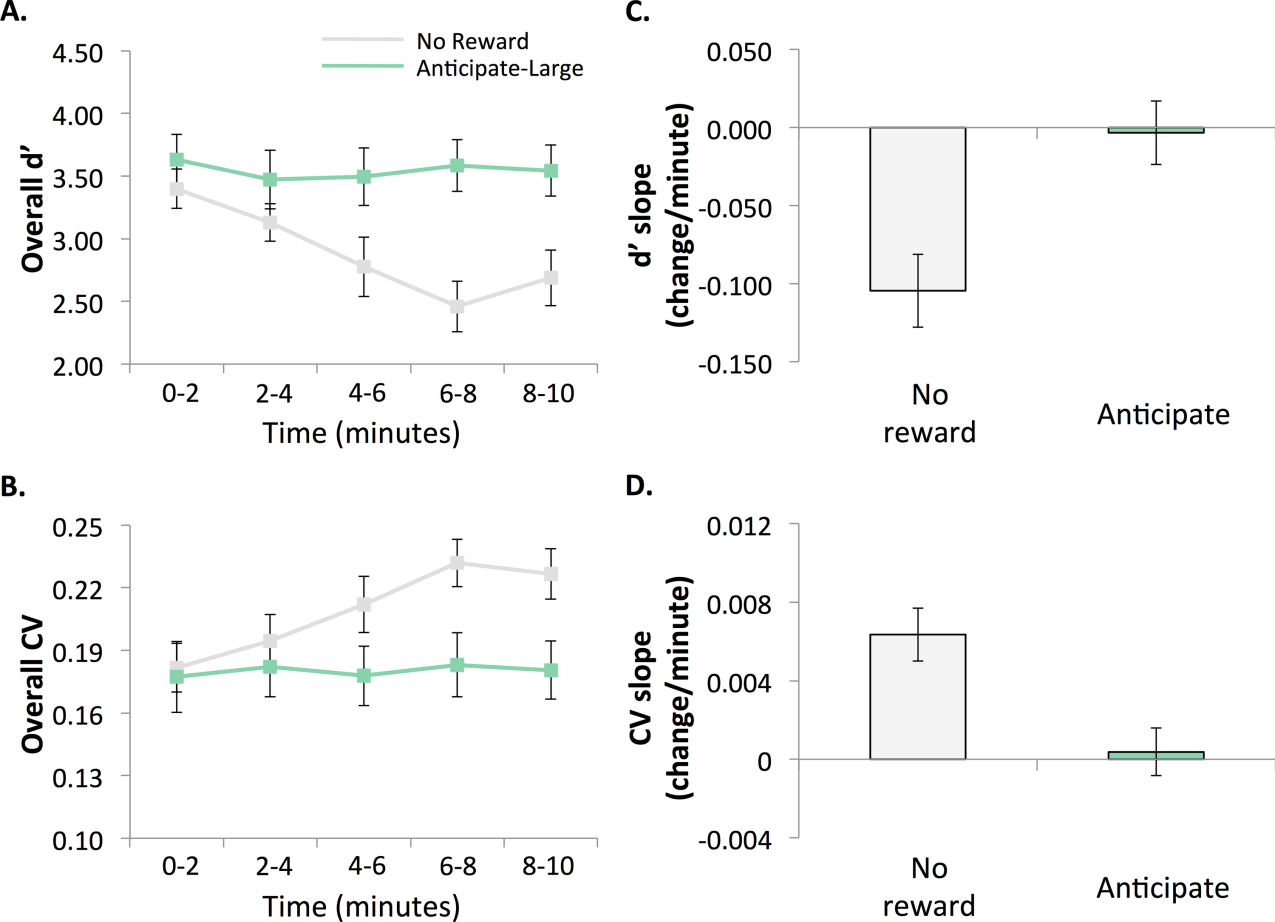
Performance in Experiment 2 divided into 2-min quintiles. A. Accuracy (d’) and B. RT variability (CV). **Vigilance decrements, or linear changes over time, in Experiment 2**. C. Accuracy (d’) and D. RT variability (CV). Significant decrements were observed in the No-reward task, but not for the Anticipate-large-loss task.

In the no-loss (practice) version of gradCPT, there was a vigilance decrement in d’ (M = -0.105, SD = 0.099) and this slope was significantly different from 0 (t(17) = -4.475, *p*<0.001, η^2^ = .54). However, during the subsequent anticipate-large-loss task, there was no decrement (M = -0.0033, SD = 0.0867) and the slope was not significantly different from 0 (t(17) = -0.162, *p* = 0.873, η^2^ = .0015) and was not significantly different from the anticipated-large-loss task in Experiment 1a (t(34) = 0.299, *p* = 0.767, η^2^ = .0026). See Fig 4A and 4C.

#### Reaction time variability

Similar to accuracy, there was a decrement in RT variability during the non-rewarded condition (M = 0.0064, SD = 0.00573) and the slope was significantly different from 0 (t(17) = 4.706, *p*<0.001, η^2^ = .57). However, during the subsequent anticipate-large-loss task, there was no decrement in RT variability (M = 0.0004, SD = 0.00515) and this slope was not significantly different from 0 (t(17) = 0.305, *p* = 0.764, η^2^ = .0054) or the anticipate-large-loss task in Experiment 1a (t(34) = 0.026, *p* = 0.980, η^2^ = .000020). See Fig 4B and 4D.

These results replicate and extend Exp. 1a, showing that when the anticipate-large-loss task was performed second, following a non-rewarded gradCPT, there was no decrement in accuracy or variability.

## Methods: Experiment 3

### Participants

Eighteen participants recruited from the same two local universities as in Experiment 1 and 2 (10 females) ages 19–30 (M = 22.27, SD = 2.67) performed the anticipate-large-loss task twice. The study was approved by the VA Boston Healthcare System IRB, and written consent was obtained from all participants.

### Procedure

The procedure in Experiment 3 was identical to Experiment 1, except that the participants completed the anticipate-large-loss task twice, instead of performing both an anticipate-large-loss and a continuous-small-loss version. This design was used to keep the context consistent throughout the whole experiment to see if the difference in context from Experiment 1a and Experiment 2 affected the vigilance decrement.

## Results: Experiment 3

### Overall performance

#### Accuracy

Participants’ overall accuracy (*d’)* was 3.05 (SD = 0.742) for the first anticipate-large-loss task and 3.36 (SD = 0.968) for the second anticipate-large-loss task. There was not a significant difference between them (t(34) = -1.08, *p* = 0.219, η^2^ = .033).

#### Reaction time variability

The overall RT variability in the first and second task was 0.196 (SD = 0.0371) and 0.188 (SD = 0.0566), respectively. There was not a significant difference between the two tasks (t(34) = 0.501, *p* = 0.619, η^2^ = .0073).

### Time-on-task effects

#### Accuracy

**Fig 5:**
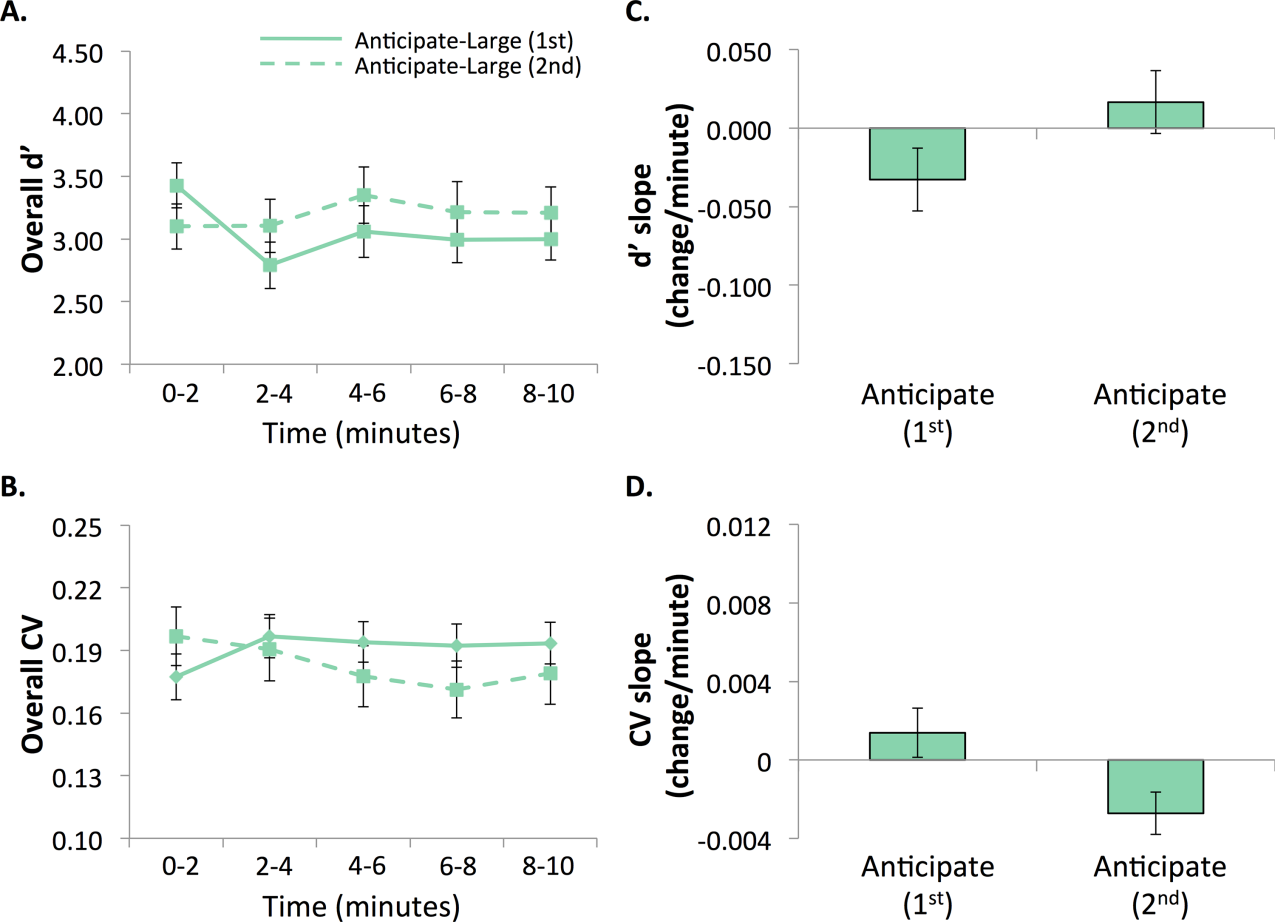
Performance in Experiment 3 divided into 2-min quintiles. A. Accuracy (d’) and B. RT variability (CV). **Vigilance decrements, or linear changes over time, in Experiment 3**. C. Accuracy (d’) and D. RT variability (CV). No significant decrements were observed in either the first or second Anticipate-large-loss task.

We replicated Experiment 1a and Experiment 2, that when performed second, the anticipate-large-loss task elicited no decrement in accuracy (M = 0.0165, SD = 0.085; not significantly different from 0, t(17) = 0.825, *p* = 0.421, η^2^ = .039) (Fig 5A and 5C). Interestingly, we also found that when performed first, there was also no decrement in accuracy (M = -0.0328, SD = 0.085, not significantly different from 0, t(17) = -1.635, *p* = 0.12, η^2^ = .14). In addition, neither condition was significantly different than anticipate-large loss in 1a (first time: t(34) = -0.823, *p* = 0.416, η^2^ = .020; second time: t(34) = 1.06, *p* = 0.297, η^2^ = .032), but were significantly different from the anticipate-large loss in 1b (first time: t(33) = 2.16, *p* = 0.038, η^2^ = .12, second time: t(33) = 3.628, *p* = 0.001, η^2^ = .29).

#### Reaction time variability

A similar decrement pattern was seen in RT variability (Fig 5B and 5D). Similar to accuracy, neither first nor second anticipate-large-loss tasks exhibited decrements in variability. The first anticipate-large-loss task had no significant decrement (M = 0.0014, SD = 0.0053, not significantly different from 0, t(17) = 1.108, *p* = 0.283, η^2^ = .067). For the second anticipated-large-loss task, the slope was significantly different from 0, but this is because the participants showed *improvement* over the course of the task (M = -0.0027, SD = 0.0046; t(17) = -2.523, *p* = 0.022, η^2^ = .27). Furthermore, the first task was not significantly different than the anticipated-large loss in Experiment 1a (where this condition came second, t(34) = 0.704, *p* = 0.486, η^2^ = .014), but the second task was, again because the participants in Experiment 3 decreased (improved) RT variability over time during the second task (t(34) = -2.255, *p* = 0.031, η^2^ = .13). Compared to anticipate-large loss in 1b, the first anticipate-large loss did not reach significance (t(33) = -1.654, *p* = 0.108, η^2^ = .077) but the second anticipate-large loss did (t(33) = -4.293, *p*<0.001, η^2^ = .36).

Taken together, we found in 3 datasets that decrements in accuracy and variability were eliminated when the anticipate-large loss is completed second. This demonstrates that practice, rather than context, is the more likely explanation for the interaction with order in Experiment 1. However, we also observed a lack of performance decrements *even* in the first anticipate-large-loss task in Experiment 3. This does not replicate the results from Experiment 1b, and leaves open the possibility that when performed first, a vigilance decrement may still be present in some subjects (Exp. 1b), but to a more variable degree. Note that in Experiment 3, when performed first, there was a numerical decrement in accuracy and increase in variability, but only after the first quintile, followed by 8 minutes of relative stability.

## Discussion

The results of the current study provide insight into the nature of vigilance decrements that accompany sustained attention tasks. In three experiments that varied the motivation and the order of the tasks, we sought to determine how prior experience and type of reward could reduce these decrements. We demonstrate that holding the motivation constant throughout the task was able to eliminate the vigilance deficits over time. However, this motivational effect was most consistently observed with prior task experience (Experiments 1a, 2, and 3), with mixed results when performed without task experience (Experiment 1b vs. 3). Our results suggest that the most reliable way to attenuate the decrement is with an anticipated-large loss looming, particularly when performed after a prior 10-minute vigil with the task. These findings have novel implications for theoretical models of sustained attention as well as potential practical relevance.

By showing that in certain circumstances the vigilance decrement can be reduced/eliminated, the current results have important theoretical and practical implications for improving sustained attention. There have been several attempts to improve sustained attention, such as through cognitive training (e.g., [33]), meditation (e.g., [34]), and the implementation of breaks [35] (although see [36]). The current study is unique in that we were able to attenuate the vigilance decrement rather than simply modulate overall performance (although see [37]). We most consistently demonstrated this attenuation when the task was practiced. One potential implication is that for well-practiced, relatively shorter-duration tasks like those in the current experiment (e.g., security baggage screening, radiologist reading scans for cancer, checking passports), it is possible to reduce performance decrements by having an unpredictable, forthcoming high reward. It would be informative to extend the current results to these more real world settings and determine if the anticipation of an impending reward or punishment could also attenuate the vigilance decrement in such contexts.

Besides demonstrating a novel way of improving sustained attention, the current results also have theoretical implications for models of sustained attention. The opportunity-cost model by [23] posits that vigilance decrements during sustained attention tasks are due to reallocation (rather than depletion) of cognitive resources. In contrast to resource accounts which attribute performance decline to depletion of limited cognitive resources [16], the opportunity cost model argues that cognitive mechanisms are allocated to those tasks carrying the greatest benefit relative to the perceived cost. Previously, our group [18] demonstrated that sustained attention tasks with continous-small rewards fail to attenuate vigilance decrements, despite improving overall performance. The current data provide a replication of these findings, which are in line with both resource and opportunity cost accounts (Experiments 1a and 1b). Importantly however, our current finding that holding reward opportunity constant (with the impending large-loss trial) can eliminate the vigilance decrement disputes a purely resource account. Instead, we suggest that in typical continuous-small-reward, or trial-to-trial reward tasks, the potential for future rewards decreases over time, and as the task proceeds the perceived effort/cost of task completion exceeds the potential benefits. This motivates reallocation of resources to activities perceived as more rewarding and lower effort (e.g., mind wandering), resulting in performance decline (vigilance decrement). In our anticipate-large-loss version however, the fixed opportunity for large reward (i.e., $18) exceeds the perceived value of task-unrelated mental activities, and motivates the individual to continue allocating their cognitive resources to the current task.

The mixed results of the anticipate-large-loss task when performed first (Exp. 1b vs. Exp. 3), however indicate that the motivational differences cannot completely account for the vigilance decrements. In Experiment 1b, despite having some practice trials before the first task vigil (i.e., 1–2 minutes), participants still exhibited a vigilance decrement. Even in Experiment 3, when performed first, there was a hint of vigilance decrement initially (1^st^ to 2^nd^ quintile), suggesting that consistent performance over time may require some practice. Together, these results suggests that there exist additional information processing demands in novel tasks, which may deplete the resources needed to maintain vigilant performance. Attention research has often considered the effect of novelty itself to be protective of lapses (e.g., [38]), and habituation/practice is typically thought to increase attention failures (e.g., [4]), On the other hand, novel tasks may be more arousing, and arousal has been shown to exacerbate the vigilance decrement under some circumstances [39]. Similarly, novel tasks may require more working memory demands (e.g.,[32]), and increasing working memory demands can increase vigilance decrements [22]. Thus, resource models [16] as well as the strength model of self-control [40, 41] might predict that novel challenging and/or arousing tasks could deplete a global resource in a qualitatively different way than practiced tasks, as they may require more controlled processing and activation of task-positive brain networks (e.g., [38, 42]; although see [43] for a more complex pattern). We further speculate that after 10-minutes of experience, participants may be able to avoid potential resource depletion if they are aware of the duration and difficulty of the task at hand, as demonstrated by the results of the anticipated-large-loss task performed second. This explanation still fits with the opportunity cost model [23] because the decision to allocate resources and prevent depletion could require prior experience of the costs and benefits of doing the task (in the first vigil).

An alternative interpretation of the anticipated-large loss vigilance decrement when completed first comes from the examination of the starting (first quintile) of performance in Experiment 1b, in that these subjects were particularly good at the task initially and may have had more room to drop off in performance. While this may suggest that better initial performance may generally make one more susceptible to vigilance decrements, we confirmed that this could not account for the lack of decrement in the three experiments with the anticipate-large-loss task performed second. Specifically, these participants (Exp. 1a, Exp. 2, Exp. 3) had significantly smaller decrements than those who did the incremental-loss version second (Exp. 1b), even when controlling for first quintile performance (p<0.02).

There are a number of limitations in the current studies. Firstly, the tasks used were of shorter duration than many traditional vigilance tasks and therefore decrements in the anticipate-large loss could have emerged with longer time on task. Although one study of the vigilance decrement demonstrated that subjects were able to maintain attention for over two hours [37], it is difficult to compare the endurance of attention across tasks [25]. Nonetheless, vigilance decrements observed in high-load short tasks have been shown to behave similarly and engage the same brain networks as those in more lengthy tasks, suggesting a common mechanism [28, 44]. Moreover, our finding that perceived opportunity cost is able to attentuate performance decline, contrasted with the reliable vigilance decrements in tasks of the same duration both in the current and previous studies, strongly suggests that resource explanations alone cannot account for our findings.

Another concern is that different types of continuous performance tasks may measure different types of sustained attention. Traditional X-CPTs are typically longer and require responses to infrequent stimuli. Not-X CPTs however are often shorter in duration and require responses to the majority of stimuli, withholding on a few rare trials. Where X-CPTs often measure aspects of sustained perceptual sensitivity, not-X CPTs more so measure attentional and response control (see [28] for discussion). Despite this, vigilance decrements in different types of task have been shown to have similar properties both behaviorally [45] and in terms of functional brain imaging [46]. Further research however should investigate whether our findings in a challenging go/nogo task requiring frequent responses extend to other types of vigilance tasks and as such other aspects of sustained attention.

Given the relatively small number of subjects, we did not evaluate individual differences in performance. It could be that individuals with high reward sensitivity, grit, or lower boredom proneness are more likely to stay on task. Such individual difference studies could help elucidate cognitive and neural interactions between reward and attention. For example, [47] found that while reward leads to sustained increases in right-lateralized frontal-parietal control regions, individuals with high reward sensitivity had additional activity in frontopolar and reward-sensitive brain regions. Along these lines, concurrent neuroimaging characterizing individual differences is also a potential fruitful future direction.

The current study provides important insights into resolving conflicting models of sustained attention failures. Previously, we demonstrated that incremental reward was unable to attenuate vigilance decrements, lending support to a resource depletion account [18]. The current study extends our understanding by demonstrating that contrary to depleting resources, holding opportunity cost constant enabled participants to overcome typical performance decline. Our finding that this only occurs consistently with sufficient task experience opens up avenues of future research into the interaction between perceived benefit/costs and practice. In addition, resource versus motivation models have several implications beyond sustained attention [23]. The notion that performance decline is a consequence of benefit/cost decisions rather than an inevitability is applicable to many executive functions that require recruitment of cognitive resources with the potential to be allocated to different tasks. Along these lines, our results have potential implications for diverse but related literatures such as self-control [48], grit [49], and ADHD (e.g., [50]). The novel methods developed in the current study may inform future work in these diverse research areas.
